# 2346. Preliminary results of durable immune response induced by a self-amplifying mRNA (samRNA) SARS-CoV-2 vaccine candidate, GRT-R910, in adults previously vaccinated with mRNA or AZD1222 primary series

**DOI:** 10.1093/ofid/ofad500.1968

**Published:** 2023-11-27

**Authors:** Alison Uriel, Pedro Garbes, A Nagare, J C Kuan, Christine Palmer, K Bae, Karin Jooss, A Rappaport, Melissa Kachura, L Kraemer, Harshni Venkatraman, Jason Jaroslavsky, Sonia Kounlavouth, M Marrali, Meghan Hart, J Betular, A Allen, C Green, O Osanlou, Adrian Palfreeman, Tolga Turgut, Andrew Ustianowski

**Affiliations:** National Institute of Health Research Manchester, Manchester, England, United Kingdom; Gritstone bio, Inc., Weston, Massachusetts; Gritstone bio, Inc, San Jose, California; Gritstone Bio, Cambridge, Massachusetts; Gritstone Bio, Cambridge, Massachusetts; Gritstone bio, Inc, San Jose, California; Gritstone bio, Inc., Weston, Massachusetts; Gritstone bio, Inc, San Jose, California; Gritstone Bio, Cambridge, Massachusetts; Gritstone bio, Inc, San Jose, California; Gritstone Bio, Cambridge, Massachusetts; Gritstone Bio, Cambridge, Massachusetts; Gritstone Bio, Cambridge, Massachusetts; Gritstone bio, Inc, San Jose, California; Gritstone Bio, Cambridge, Massachusetts; Gritstone bio, Inc, San Jose, California; Gritstone bio, Inc, San Jose, California; University Hospitals Birmingham NHS Foundation Trust - Birmingham (United Kingdom), Birmingham, England, United Kingdom; North Wales Clinical Research Facility - North Wales (United Kingdom) and Bangor University – North Wales (United Kingdom), North Wales, Wales, United Kingdom; University Hospitals of Leicester NHS Trust, Leicester, England, United Kingdom; National Institute of Health Research Manchester, Manchester, England, United Kingdom; North Manchester General Hospital, Manchester, England, United Kingdom

## Abstract

**Background:**

Neutralizing antibodies (nAbs) induced by authorized SARS-CoV-2 vaccines wane within months, requiring frequent boosters. Preliminary safety and immunogenicity of a samRNA-based SARS-CoV-2 vaccine candidate (GRT-R910) administered as a booster following AZD1222 primary series in older (≥ 60 years) adults (Cohorts 1 & 2, CORAL-BOOST study, NCT05148962) were previously reported. Updated results of GRT-R910 administered as a 2-dose booster in older (≥ 60 years) adults who received a primary series of either AZD1222 or mRNA vaccines (Cohorts 3 (C3) & 4 (C4)) are now reported.

**Methods:**

GRT-R910 encodes Wuhan Spike (S) and highly conserved non-S T cell epitopes, is being tested in this open-label phase I study, conducted in the UK. Two booster doses of 10µg GRT-R910 are assessed in older adults who have received (C3) or mRNA vaccines (C4) as primary series. Nine participants (C3) received an mRNA booster prior to enrollment. The primary objective is safety and secondary objectives include assessment of titers and durability of S-specific IgG (bAbs) and nAb responses of GRT-R910 boost vaccinations as well as T cell responses to S and non-S epitopes.

**Figure:**
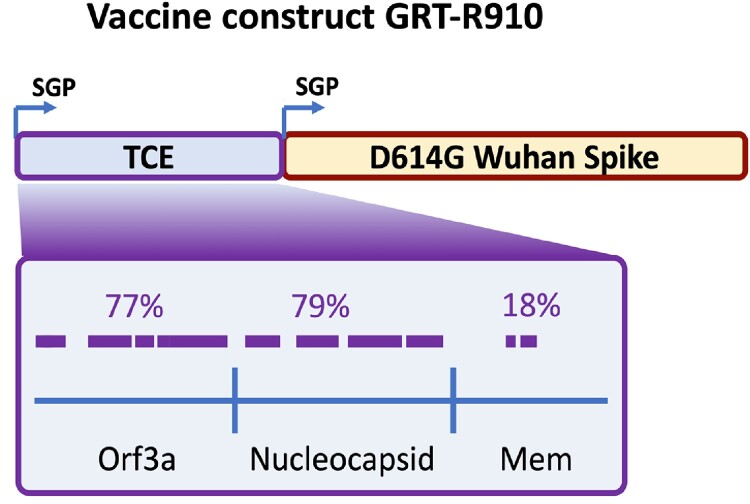

**Figure:**
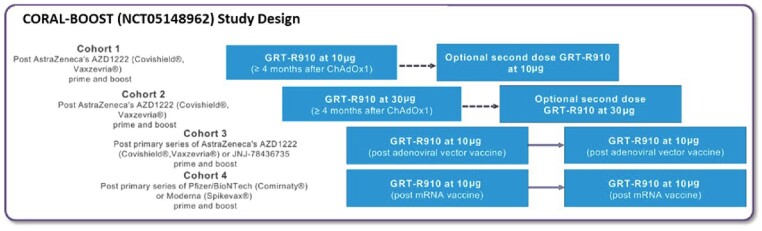

**Results:**

Ten participants were enrolled in each cohort (C3 and C4). The majority of reactogenicity events were mild or moderate and transient in nature. Three subjects reported grade 3 solicited adverse events which resolved within 1-4 days. Local and systemic reactogenicities were milder with the 2^nd^ booster compared to the 1^st^ booster across all cohorts. All participants showed bAbs IgG > 500 ELU/mL and nAbs against to the vaccine-specific strain and nAbs > 500 ND_50_ at 6 months following GRT-R910 vaccination, regardless of age, primary vaccination series, or additional prior mRNA booster. T cell responses to S were boosted and maintained up to at least Day 57; analyses of non-S T cell responses are ongoing.

**Conclusion:**

GRT-R910 was well tolerated as a booster in older adults regardless of primary SARS-CoV-2 vaccination series. The 2^nd^ booster was less reactogenic than the 1^st^ and less reactogenicity was observed in the older population. High and sustainable antibody responses were observed for at least 6 months against Wuhan Spike across different age groups. Data on other variants will also be presented.

**Disclosures:**

**Alison Uriel, MBBS**, North Manchester General Hospital, UK. - Manchester (United Kingdom): Employee **Pedro Garbes, MD**, Gritstone bio, Inc.: Employee|Gritstone bio, Inc.: Employee|Gritstone bio, Inc.: Stocks/Bonds|Gritstone bio, Inc.: Stocks/Bonds **A. Nagare, MBBS**, Gritstone bio, Inc.: Employee|Gritstone bio, Inc.: Stocks/Bonds **JC Kuan, PhD**, Gritstone bio, Inc.: Employee|Gritstone bio, Inc.: Stocks/Bonds **Christine Palmer, PhD**, Gritstone bio, Inc.: Employee|Gritstone bio, Inc.: Stocks/Bonds **K. Bae, PhD**, Gritstone bio: Stocks/Bonds **Karin Jooss, PhD**, Gritstone bio: employee|Gritstone bio: Stocks/Bonds **A. Rappaport, PhD**, Gritstone bio, Inc.: Employee|Gritstone bio, Inc.: Stocks/Bonds **Melissa Kachura, BS**, Dynavax Technologies: WO2019040491A1|Dynavax Technologies: Stocks/Bonds|Gritstone bio, Inc.: Employee|Gritstone bio, Inc.: Stocks/Bonds **L. Kraemer, PhD**, Gritstone bio, Inc.: Employee|Gritstone bio, Inc.: Stocks/Bonds **Harshni Venkatraman, MS**, Gritstone bio, Inc.: Employee|Gritstone bio, Inc.: Stocks/Bonds **Jason Jaroslavsky, MS**, Gritstone bio, Inc.: Employee|Gritstone bio, Inc.: Stocks/Bonds **Sonia Kounlavouth, BS**, Gritstone bio, Inc.: Employee|Gritstone bio, Inc.: Stocks/Bonds **M. Marrali, PhD**, Gritstone bio, Inc.: Employee|Gritstone bio, Inc.: Stocks/Bonds **Meghan Hart, ALM**, Gritstone bio, Inc.: Employee|Gritstone bio, Inc.: Stocks/Bonds **J. Betular, PhD**, Gritstone bio, Inc.: Employee|Gritstone bio, Inc.: Stocks/Bonds **A. Allen, MBBS, PhD**, Gritstone bio, Inc.: Employee|Gritstone bio, Inc.: Ownership Interest|Gritstone bio, Inc.: Stocks/Bonds **C. Green, MBBS**, University Hospitals Birmingham NHS Foundation Trust - Birmingham (United Kingdom): Employee **O. Osanlou, MBBS**, North Wales Clinical Research Facility - North Wales (United Kingdom) and Bangor University – North Wales (United Kingdom): Employee **Adrian Palfreeman, MBBS**, University Hospital of Leicester NHS Trust. - Leicester (United Kingdom): Employee **Tolga Turgut, MBBS**, North Manchester General Hospital, UK. - Manchester (United Kingdom): Employee **Andrew Ustianowski, MD, PhD**, Gilead: Honoraria|Gilead: Advisory Board|GSK: Honoraria|Janssen: Honoraria|Merck: Honoraria|Merck: Advisory Board|Sanofi: Honoraria|ViiV Healthcare/GSK: Advisory Board

